# Electrocardiographic left atrial abnormality and silent vascular brain injury: The Northern Manhattan Study

**DOI:** 10.1371/journal.pone.0203774

**Published:** 2018-10-12

**Authors:** Madeleine D. Hunter, Yeseon Park Moon, Charles DeCarli, Jose Gutierrez, Clinton B. Wright, Marco R. Di Tullio, Ralph L. Sacco, Hooman Kamel, Mitchell S. V. Elkind

**Affiliations:** 1 Department of Neurology, Vagelos College of Physicians and Surgeons, Columbia University, New York, New York, United States of America; 2 Department of Neurology, University of California-Davis, Davis, California, United States of America; 3 Office of Clinical Research, National Institute of Neurological Disorders and Stroke, National Institutes of Health, Washington, D.C., United States of America; 4 Department of Medicine, Vagelos College of Physicians and Surgeons, Columbia University, New York, New York, United States of America; 5 Department of Neurology, Miller School of Medicine, University of Miami, Miami, Florida, United States of America; 6 Departments of Public Health Sciences, Human Genetics & Neurosurgery, Miller School of Medicine, University of Miami, Miami, Florida, United States of America; 7 Department of Neurology, Weill-Cornell Medical School, Cornell University, New York, New York, United States of America; 8 Department of Epidemiology, Mailman School of Public Health, Columbia University, New York, New York, United States of America; Indiana University, UNITED STATES

## Abstract

**Hypothesis:**

We hypothesized that P wave terminal Force in the V1 lead (PTFV_1_) would be associated with leukoaraiosis and subclinical infarcts, especially cortical infarcts, in a population-based, multi-ethnic cohort.

**Methods:**

PTFV_1_ was collected manually from baseline electrocardiograms of clinically stroke-free Northern Manhattan Study participants. Investigators read brain MRIs for superficial infarcts, deep infarcts, and white matter hyperintensity volume (WMHV). WMHV was adjusted for head size and log transformed, achieving a normal distribution. Logistic regression models investigated the association of PTFV_1_ with cortical and with all subclinical infarcts. Linear regression models examined logWMHV. Models were adjusted for demographics and risk factors.

**Results:**

Among 1174 participants with PTFV_1_ measurements, the mean age at MRI was 70 ± 9 years. Participants were 14.4% white, 17.6% black, and 65.8% Hispanic. Mean PTFV_1_ was 3587.35 ± 2315.62 μV-ms. Of the 170 subclinical infarcts, 40 were cortical. PTFV_1_ ≥ 5000 μV-ms was associated with WMHV in a fully adjusted model (mean difference in logWMHV 0.15, 95% confidence interval 0.01–0.28). PTFV_1_ exhibited a trend toward an association with cortical infarcts (unadjusted OR per SD change logPTFV_1_ 1.30, 95% CI 0.94–1.81), but not with all subclinical infarcts.

**Conclusion:**

Electrocardiographic evidence of left atrial abnormality was associated with leukoaraiosis.

## Introduction

Subclinical cerebrovascular disease (SCVD) includes subclinical infarcts and leukoaraiosis, or white matter hyper-intensities often found in the peri-ventricular region but also in the subcortical white matter. [1] SCVD has been found to be associated with cognitive decline [2, 3, 4] and an increased incidence of ischemic stroke [2]. In addition, associations between SCVD and vascular risk factors have been found. For example, age and hypertension were associated with subclinical infarcts [5, 6]. Though the etiology of leukoaraiosis remains uncertain, it is generally thought to represent a manifestation of small vessel vascular disease, and some studies have found an association between progression of leukoaraiosis and presence of vascular risk factors such as current smoking, [7, 8] lower high density lipoprotein, [7] and hypertension [8], though not all studies have found each of these associations. [8, 9]

P wave terminal force in ECG lead V1 (PTFV_1_), a marker of “atrial cardiopathy” or left atrial dysfunction [10], has been associated with clinical stroke, presumably through a cardioembolic mechanism [11]. PTFV1 is thought to reflect atrial cardiopathy by indicating the presence of electrical and anatomical changes in the left atrium. It has been suggested that atrial cardiopathy could include a series of pathological changes in atria which would include the electrical and anatomical changes reflected in PTFV1 but also changes to the atrial substrate which could encourage thromboembolism [5, 12]. In addition, leukoaraiosis was found to be associated with PTFV_1_ in a predominantly white population [13].

Though the effect of PTFV_1_ on SCVD has been investigated in a predominantly white population [13], it has not been well-studied in Hispanics and blacks. We hypothesized that PTFV_1_ would be associated with an increased prevalence of subclinical infarcts, especially cortical ones that are more likely related to embolic mechanisms, and leukoaraiosis, in a population-based, multi-ethnic cohort.

## Methods

### The cohort

The Northern Manhattan Study (NOMAS) is a race/ethnically diverse population-based prospective cohort of participants free of clinical stroke at baseline who were recruited between 1993 and 2001, as previously described [14]. After a baseline in-person examination, the 3,298 participants were followed annually by telephone to determine medical, socio-economic, and other risk factors as well as the incidence of stroke and other vascular outcomes. Starting in 2003, NOMAS participants were invited to participate in an MRI substudy, which required them to 1) be older than 50 years; 2) have no contraindications to MRI; and 3) be free of a clinically recognized stroke. To increase the number of participants in the MRI study to 1,290, 199 household members of NOMAS participants were also invited to participate in the substudy. All participants gave informed consent. The study was approved by the Institutional Review Boards at Columbia University Medical Center (CUMC) and the University of Miami.

### Baseline evaluation of cohort

Participants underwent a thorough baseline examination including comprehensive medical history, physical examination, and review of medical records. Study definitions for race–ethnicity, hypertension, diabetes, cardiac disease, and other risk factors have been described previously [15]. Trained bilingual research assistants performed interviews; study physicians conducted physical and neurological examinations.

### Measurement of P wave terminal force

The predictor, PTFV_1_, is the product of the depth (μV) and duration (ms) of the terminal portion of the electrocardiogram (ECG) V1 lead’s biphasic P wave. PTFV_1_ was calculated from paper ECGs taken at the time of initial enrollment for all participants in the MRI substudy, a mean of 6.0 ± 3.4 years prior to MRI. The measurements of depth and duration were ascertained manually with the use of a digital caliper by an investigator (MDH) who was blinded to outcome. Measurements were then converted to μV and ms using the standard ECG calibration of 10 mm/mV and 25 mm/s. Prior studies have shown excellent intra-rater correlations and moderate inter-rater correlations for manual measurements of P-wave morphology [16]. To confirm this in our cohort, as previously described, a second reader independently performed blinded measurements of a random sample of 30 ECGs. Cases were excluded and PTFV_1_ was coded as missing when inadequate ECG quality or the absence of P-waves due to active AF prevented us from obtaining baseline PTFV_1_. Subjects with paroxysmal AF not active on ECG remained in the study. Intraclass correlation coefficients for PTFV_1_ measurements in the subset of 30 ECGs that were independently assessed by two investigators demonstrated excellent intra-rater reliability (0.87; 95% CI, 0.79–0.92) and moderate inter-rater reliability (0.69; 95% CI, 0.45–0.80), as previously reported [11].

### Magnetic Resonance Imaging (MRI) outcomes

The primary outcomes were subclinical infarcts and white matter hyperintensities seen on MRI. The MRI scans in NOMAS were performed according to specifications previously described [5, 17]. Subclinical infarctions were defined as a hypointensity measuring greater than 3 mm on FLAIR with no correlating signal void on T2; subclinical infarctions were also categorized as superficial or deep [18].

As previously described [19], analyses for WMHV were performed using semi-automated measurements of pixel distributions and mathematical modeling of pixel-intensity histograms for cerebrospinal fluid and brain (white and gray matter) to identify the optimal pixel-intensity threshold to distinguish cerebrospinal fluid from brain matter. Analyses were performed using a custom-designed image analysis package (QUANTA 6.2 using a Sun Microsystems Ultra 5 workstation). WMHV, which conformed to the STRIVE criteria [20], was calculated after correcting for total cranial volume to correct for differences in head size, and log-transformed to achieve a normal distribution (log-WMHV) for analysis. All analyses were performed blinded to participant identifying information and biomarker measurements.

### Statistical analysis

Distributions of baseline characteristics were calculated. The predictor of interest, PTFV_1_ was log transformed to achieve linearity and we used standard deviation (SD) increments as a continuous measure. PTFV_1_ was also analyzed by quartiles and using a threshold used in prior studies and also previously shown to be associated with risk of clinical stroke (>5000 μV*ms) [21, 22, 23]. Logistic regression models were used to calculate odds ratios (OR) and 95% confidence intervals (CI) for the association of PTFV_1_ with both all subclinical infarcts and with superficial subclinical infarcts. Linear regression was used for the association of PTFV_1_ with WMHV. Adjusted models accounted for demographics (race-ethnicity, age, sex, insurance status, education) and risk factors (atrial fibrillation, congestive heart failure, diabetes, diastolic and systolic blood pressure, smoking, high and low density lipoprotein cholesterol, glomerular filtration rate). In a subsample in which left atrial diameter was available (n = 958), additional analyses were performed further adjusting for left atrial diameter and left atrial enlargement. All statistical analyses were performed using SAS version 9.3 (SAS Institute, Cary, NC).

## Results

### Description of the cohort

Among the 1174 participants with MRI scans and PTFV_1_ available, the mean age was 70 ± 9 years at the time of MRI, 40.3% were male, and 14.4% were white, 17.6% black, and 65.8% Hispanic. Most (68.0%) of participants had hypertension. MRIs were performed a mean of 6.0 ± 3.4 years after ECG. Subclinical infarcts were present in 170 participants (15.1%), and were superficial in 40 (3.6%). PTFV_1_ had a mean of 3587.35 ± 2315.62 μV-ms and a median of 3063 μV-ms (interquartile range 2029.6–4559.2). The proportion of PTFV_1_>5000 μV-ms was 20%. Among the 958 participants with left atrial diameter available, the mean diameter was 36.0 ± 4.7 mm. According to categories used in previous studies, 80.3% had normal left atrial size (women, ≤38 mm; men, ≤40 mm), and 26 had moderate (women, 43–46 mm; men, 47–51 mm) or severe left atrial enlargement (women, ≥47 mm; men, ≥52 mm) [24]. Further baseline characteristics are reported in Table 1.

**Table 1:** Baseline characteristics of the Northern Manhattan Study Magnetic Resonance Imaging cohort (n = 1174).

Socio-demographic Characteristics	Mean ± SD or n (%)
Age, years	70 ± 9
Women	701 (59.7)
Race-Ethnicity	
Hispanic	773 (65.8)
Non-Hispanic Black	207 (17.6)
Non-Hispanic White	169 (14.4)
Less than high school education	638 (54.3)
Medicaid or no insurance	555 (47.3)
Medical Characteristics	
Smoking Status	
Never used	559 (47.6)
Former smoker	429 (36.5)
Current user	186 (15.8)
Hypertension	798 (68.0)
Systolic blood pressure (mmHg)	139 ± 20
Diastolic blood pressures in mmHg	82.7 ± 10.6
Congestive heart failure	33 (2.81)
Low-density lipoprotein in mg/dl	128.0 ± 35.2
High-density lipoprotein in mg/dl	46.8 ± 14.7
Diabetes mellitus	227 (19.3)
Estimated Glomerular filtration rate in mL/min/1.73m^2^	80.1 ± 17.0
Left atrial diameter in mm	36.0±4.7

### Association of PTFV_1_ with subclinical infarction

There was a trend toward an association of PTFV_1_ with cortical infarcts (unadjusted OR per SD change logPTFV_1_ 1.30, 95% CI 0.93–1.81) but not with all subclinical infarcts (unadjusted OR 1.00, 95% CI 0.85–1.18) in unadjusted models. In models adjusting for demographics and all risk factors other than left atrial size, there was no longer a trend toward an association with cortical infarcts (adjusted OR 1.09, 95% CI 0.76–1.57). Subclinical infarcts remained unassociated in adjusted models (adjusted OR 0.96, 95% CI 0.80–1.15). When compared to the first quartile of PTFV_1_, the fourth quartile exhibited a trend toward an association with greater prevalence of cortical infarcts in an unadjusted model (unadjusted OR 2.73, 95% CI 0.96–7.76). The trend was attenuated when adjusted for demographics and risk factors (unadjusted OR 1.86, 95% CI 0.61–5.71). Including left atrial size in the models among those with this measure available did not materially change the results. All results for subclinical infarcts are reported in Table 2.

**Table 2:** Association between P-wave terminal force in lead V_1_ (PTFV_1_) and risk of Subclinical Cerebral Infarcts (n = 1174).

		Unadjusted OR (95% CI)	Adjusted for Demographics* OR (95% CI)	Adjusted for Demographics and Risk Factors † OR (95% CI)	Adjusted for Demographics, Risk Factors, and Left Atrial Size OR (95% CI) (n = 958)‡
Overall Subclinical Infarcts					
logPTFV_1_	per SD	1.00 (0.85–1.18)	0.96 (0.81–1.13)	0.96 (0.80–1.15)	0.94 (0.76–1.15)
PTFV_1_	**Reference**: First quartile (<2029.6 μV-ms)	ref.	ref.	ref.	ref.
	Second quartile (2029.6 μV-ms − 3063.0 μV-ms)	1.01 (0.63–1.61)	1.03 (0.64–1.68)	1.08 (0.65–1.79)	1.06 (0.60–1.87)
	Third quartile (3063.0 μV-ms − 4559.2 μV-ms)	1.05 (0.66–1.67)	1.06 (0.65–1.72)	1.04 (0.62–1.73)	1.07 (0.61–1.88)
	Fourth quartile (>4559.2 μV-ms)	1.14 (0.72–1.81)	1.01 (0.62–1.64)	1.04 (0.63–1.74)	0.87 (0.48–1.57)
PTFV_1_	**Reference**: <5000 μV-ms	ref.	ref.	ref.	ref.
	>5000 μV-ms	1.19 (0.80–1.77)	1.00 (0.65–1.52)	1.02 (0.66–1.57)	0.91 (0.54–1.51)
Subclinical Cortical Infarcts					
logPTFV_1_	per SD	1.30 (0.93–1.81)	1.19 (0.85–1.65)	1.09 (0.76–1.57)	1.03 (0.69–1.52)
PTFV_1_	**Reference**: First quartile (<2029.6 μV-ms)	ref.	ref.	ref.	ref.
	Second quartile (2029.6 μV-ms − 3063.0 μV-ms)	2.21 (0.76–6.44)	2.24 (0.75–6.62)	2.20 (0.71–6.75)	2.01 (0.64–6.37)
	Third quartile (3063.0 μV-ms − 4559.2 μV-ms)	2.29 (0.79–6.68)	2.20 (0.74–6.53)	1.74 (0.56–5.43)	1.43 (0.44–4.62)
	Fourth quartile (>4559.2 μV-ms)	2.73 (0.96–7.76)	2.27 (0.77–6.64)	1.86 (0.61–5.71)	1.30 (0.40–4.23)
PTFV_1_	**Reference**: <5000 μV-ms	ref.	ref.	ref.	ref.
	>5000 μV-ms	1.55 (0.77–3.17)	1.22 (0.58–2.58)	1.10 (0.50–2.39)	0.84 (0.34–2.07)

Subclinical cortical infarcts exhibited trends to an association with log PTFV_1_, the fourth quartile of PTFV_1_, and a PTFV_1_ measurement above 5000 μV-ms which is a threshold that has been used in previous studies. However, each of these trends were attenuated with adjustments and no significant associations were found.

*Demographics include: race-ethnicity, age, sex, insurance status, and education

^†^ Risk factors include: baseline atrial fibrillation, congestive heart failure, diabetes, diastolic and systolic blood pressure, smoking, low and high-density lipoprotein cholesterol, and glomerular filtration rate

^‡^ Excluded subjects did not have left atrial size measurements

### Association of PTFV_1_ with WMHV

PTFV_1_ >5000 μV-ms was associated with greater WMHV than PTFV_1_ <5000 μV-ms in an unadjusted model (mean difference in logWMHV 0.28, 95% CI 0.14–0.42). The association remained after adjusting for demographics and risk factors except left atrial diameter (mean difference in logWMHV 0.15, 95% CI 0.01–0.28). Among the 958 participants with left atrial size available, when left atrial diameter was included as a covariate, the association was no longer statistically significant (mean difference in logWMHV 0.14, 95% CI -0.02–0.29).

When examined by quartiles, only the fourth quartile of PTFV_1_ (vs. the first quartile) was associated with greater WMHV in the unadjusted model (mean difference in logWMHV 0.25, 95% CI 0.09–0.41). Similarly, PTFV_1_ as a continuous measure was associated with WMHV in the unadjusted model (mean difference in logWMHV per SD of logPTFV_1_ 0.08, 95% CI 0.026–0.14). The association was attenuated and lost significance when demographics and risk factors were added to the final model (mean difference in logWMHV per SD of logPTFV_1_ 0.02, 95% CI -0.03–0.07). Further adjusting for left atrial size did not change those results. These results are summarized in Table 3.

**Table 3:** Association of P-wave terminal force in lead V_1_ (PTFV_1_) with white matter hyperintensity volume (n = 1172).

		Unadjusted β* (95% CI)	Adjusted for Demographics† β* (95% CI)	Adjusted for Demographics and Risk Factors ‡ β* (95% CI)	Adjusted for Demographics, Risk Factors, and Left Atrial Size β* (95% CI) (n = 958)§
logPTFV_1_	per SD	0.082 (0.03–0.14)	0.038 (-0.014–0.089)	0.019 (-0.034–0.071)	0.018 (-0.042–0.078)
PTFV_1_	**Reference**: First quartile (<2029.6 μV-ms)	ref.	ref.	ref.	ref.
	Second quartile (2029.6 μVms − 3063.0 μV-ms)	0.096 (-0.06–0.26)	0.059 (-0.086–0.20)	0.036 (-0.11–0.18)	0.026 (-0.14–0.19)
	Third quartile (3063.0 μVms − 4559.2 μVms)	0.075 (-0.086–0.23)	0.037 (-0.11–0.18)	0.010 (-0.14–0.16)	0.046 (-0.11–0.21)
	Fourth quartile (>4559.2 μVms)	0.25 (0.088–0.41)	0.13 (-0.01–0.28)	0.085 (-0.064–0.23)	0.074 (-0.094–0.24)
PTFV_1_	**Reference**: <5000 μV-ms	ref.	ref.	ref.	
	>5000 μV-ms	0.28 (0.14–0.42)	0.18 (0.050–0.31)	0.15 (0.014–0.28)	0.14 (-0.017–0.29)

In a model adjusting for demographics and risk factors except left atrial size, white Matter Hyperintensity Volume, a measure of leukoariosis, is associated with PTFV_1_ above 5000 μV-ms, a threshold used in previous studies. However, the association lost significance after adjusting for left atrial size.

* β is the average difference in logWMHV.

^†^ Demographics include: race-ethnicity, age, sex, insurance status, and education

^‡^ Risk factors include: baseline atrial fibrillation, congestive heart failure, diabetes, diastolic and systolic blood pressure, smoking, low and high-density lipoprotein cholesterol, and glomerular filtration rate

^§^ Excluded subjects did not have left atrial size measurements

## Discussion

Electrocardiographic evidence of left atrial abnormality, in our cohort, was associated with white matter hyperintensity volume, while the association with cortical infarcts seen in a previous study was not confirmed. Electrocardiographic abnormality reflected in the V1 lead through P Wave Terminal Force (PTFV_1_) is thought to reflect structural or other abnormalities of left atrial tissue, a condition that has been referred to as “atrial cardiopathy” [10]. Our finding of an association of PTFV_1_ with small vessel disease provides evidence that electrocardiographic abnormalities reflecting structural or other abnormalities of left atrial tissue may be associated with small vessel disease, cerebral hypoperfusion-related injury, or risk factors shared between small vessel disease and left atrial abnormality. However, though previous studies have suggested that atrial cardiopathy may be a source of emboli [11, 13], we did not find a statistically significant association between PTFV_1_ and superficial infarcts, which we assumed to be more likely embolic when compared to deep infarcts. Additionally, our results were attenuated in secondary analyses adjusting for left atrial size among those in whom the measurement was available, suggesting that this electrocardiographic measure of P wave abnormality may not be independent of echocardiographic measures and may reflect left atrial size. These secondary analyses, however, may have been underpowered due to the limited availability of left atrial diameter measures in the cohort.

The potential association between PTFV_1_ and WMHV is intriguing. This association was also found in a prospective cohort study done within the Cardiovascular Health Study (CHS) [13]. In the CHS study, PTFV_1_ measures were collected at baseline and repeat MRI scans five years apart were interrogated for cortical infarcts and leukoaraiosis. The association found in both studies could originate either from changes in cardiac function reflected in atrial cardiopathy or from risk factors shared by both atrial cardiopathy and leukoaraiosis. Atrial dysfunction reflected in PTFV_11_ could compromise perfusion to the brain sufficiently to damage vulnerable areas of the brain. The possibility of decreased perfusion to vulnerable areas of the brain in the setting of impaired cardiac function has previously been posited as a possible mechanism tying the association between leukoaraiosis and impaired ventricular function in patients with and without congestive heart failure. [25, 26] In prior analyses from NOMAS, we found that different left ventricular geometries and measures of left ventricular function more sensitive than ejection fraction were associated with leukoaraiosis, even in patients with normal left ventricular ejection fraction. For example, of four left ventricular geometric patterns (normal geometry, concentric remodeling, eccentric hypertrophy, and concentric hypertrophy), concentric hypertrophy was associated with leukoaraiosis, independent of ejection fraction. [27] In another analysis, another measure of subclinical cardiac dysfunction, global longitudinal strain (GLS), measured using speckle tracking on the echocardiogram, was associated with leukoaraiosis, even in patients with normal ejection fraction. Lower GLS was associated with greater white matter hyperintensity volume (adjusted β = 0.11, P<0.05). [28] In patients without congestive heart failure, Shimizu *et al* [26] found an association between diastolic dysfunction and leukoaraiosis.

Other studies, however, failed to confirm an association between cardiac output and leukoaraiosis [29], indicating that shared risk factors could be important contributors to the development of both atrial cardiopathy and leukoaraiosis. For example, increased diastolic blood pressure could contribute to leukoaraiosis and damage to the atrium as well. In fact, within NOMAS, increased diastolic blood pressure has been associated with leukoaraiosis [30]. Additionally, a prior study demonstrated the association between electrocardiographic atrial abnormalities and hypertension [31]. These observations indicate that hypertension may indeed be a shared risk factor for both atrial cardiopathy and leukoaraiosis. Though we controlled for several known shared risk factors, leukoaraiosis is incompletely understood [32], and we may not have controlled for shared risk factors that have not yet been discovered. In addition, we cannot exclude the possibility of residual confounding due to misclassification or measurement error. It is also possible that leukoaraiosis is due to cardiac emboli. Atrial fibrillation, the paradigmatic disorder causing cerebral emboli, can also be associated with white matter lesions. [33] Further studies are needed to determine whether elevated PTFV_1_ is associated with sufficient reduction in cardiac output to contribute to leukoaraiosis, and whether any such changes are independent of conventional risk factors.

Given the results of previous studies, we expected to see an association between electrocardiographic abnormality and cortical infarcts. However, the lack of statistically significant results is not surprising given the imperfect measure and small number of events. In a prior case-cohort analysis conducted in our prospective NOMAS cohort, PTFV_1_ was more strongly associated with cardioembolic and cryptogenic ischemic stroke subtypes than with ischemic stroke in general [11]. This suggests that atrial cardiopathy as ascertained on ECG may be associated with cardiac thromboembolism and thus ischemic stroke. In the analysis done on repeat MRI scans within Cardiovascular Health Study (CHS), PTFV_1_ was associated with MRI-defined incident infarcts, especially superficially located, embolic-appearing events [13]. The results of the CHS study suggested again that electrocardiographic abnormality could reflect a thromboembolic mechanism which may result in ischemic stroke. This present study did not confirm the association between superficial infarcts and PTFV_1_ found in the CHS study, though the trends were often in the expected direction. It is possible that no association was found because non-differential misclassification in blinded manual measurements (“noise” or imprecision in measurements”) biased the results toward the null and the small number of superficial infarcts (n = 40) limited power. Further studies are warranted to confirm an association between PTFV_1_ and subclinical cortical infarcts.

These results should be considered within the limitations of the study. First, measurement of PTFV_1_ is subject to measurement error due to placement of the V1 lead of the ECG. Since the recording varies depending where the lead is placed, variations in lead placement will change the tracing, including the depth and width of the terminal P wave. However, this measure has been associated with outcomes in previous studies, and non-differential misclassification is likely to bias results towards the null. In a prior study conducted in the Northern Manhattan Study, moreover, which used the same ECGs, PTFV_1_ was associated with clinical ischemic stroke [11]. Also, measurements for depth and duration for the calculation of PTFV_1_ were made manually. However, measurements were made blinded to outcome, so the results were again likely biased toward the null and the true association is thus likely to be stronger. Second, the small number of subclinical infarcts limited power. Third, it is possible that not all participants with AF were identified since long-term heart-rhythm monitoring, which has been shown to be sensitive for the detection of AF, was not conducted [34]. Also, only participants with paroxysmal AF could be included, because it is not possible to collect P wave measurements during active AF. However, the models did adjust for known AF as a covariate and other studies have shown that PTFV_1_ is associated with clinical ischemic stroke even after the exclusion of participants with diagnosed AF [11].

This study also has strengths, including the race/ethnically diverse composition of the cohort as well as the method of measurement used to quantify WMHV. The Northern Manhattan Study is a prospectively enrolled population-based cohort. The cohort includes blacks and Hispanics, who are often understudied. In the MRI substudy of this cohort, volumetric techniques were used to determine white matter disease on MRI rather than often implemented semi-quantitative methods.

## Conclusion

An electrocardiographic measure of left atrial cardiopathy is associated with leukoaraiosis. This electrocardiographic measure may also be associated with subclinical cortical infarcts, though our statistical power was not sufficient to show this. Future studies should be conducted to replicate the results regarding both subclinical infarcts and leukoaraiosis in other cohorts with different demographics, and these studies should attempt to distinguish any additive value of electrocardiographic measures beyond those provided by echocardiography. Other studies should also investigate the mechanisms for the association between PTFV_1_ and WMHV. Lastly, clinical trials aimed at preventing clinical strokes with PTFV_1_ as a predictor may be warranted given the results of this and other studies. [11, 13]
